# The RNA Binding Protein RBM38 (RNPC1) Regulates Splicing during Late Erythroid Differentiation 

**DOI:** 10.1371/journal.pone.0078031

**Published:** 2013-10-18

**Authors:** Laurie A. Heinicke, Behnam Nabet, Shihao Shen, Peng Jiang, Sebastiaan van Zalen, Benjamin Cieply, J. Eric Russell, Yi Xing, Russ P. Carstens

**Affiliations:** 1 Department of Medicine (Hematology-Oncology Division), Perelman School of Medicine, University of Pennsylvania, Philadelphia, Pennsylvania, United States of America; 2 Department of Medicine (Renal Division), Perelman School of Medicine, University of Pennsylvania, Philadelphia, Pennsylvania, United States of America; 3 Department of Internal Medicine, University of Iowa, Iowa City, Iowa, United States of America; 4 Department of Microbiology, Immunology, and Molecular Genetics, University of California Los Angeles, Los Angeles, California, United States of America; 5 Department of Pediatrics (Hematology), Perelman School of Medicine, University of Pennsylvania, and the Children’s Hospital of Philadelphia, Philadelphia, Pennsylvania, United States of America; 6 Department of Genetics University of Pennsylvania, Perelman School of Medicine, Philadelphia, Pennsylvania, United States of America; Rutgers New Jersey Medical School, United States of America

## Abstract

Alternative pre-mRNA splicing is a prevalent mechanism in mammals that promotes proteomic diversity, including expression of cell-type specific protein isoforms. We characterized a role for RBM38 (RNPC1) in regulation of alternative splicing during late erythroid differentiation. We used an Affymetrix human exon junction (HJAY) splicing microarray to identify a panel of RBM38-regulated alternatively spliced transcripts. Using microarray databases, we noted high RBM38 expression levels in CD71^+^ erythroid cells and thus chose to examine RBM38 expression during erythroid differentiation of human hematopoietic stem cells, detecting enhanced RBM38 expression during late erythroid differentiation. In differentiated erythroid cells, we validated a subset of RBM38-regulated splicing events and determined that RBM38 regulates activation of Protein 4.1R (EPB41) exon 16 during late erythroid differentiation. Using Epb41 minigenes, Rbm38 was found to be a robust activator of exon 16 splicing. To further address the mechanism of RBM38-regulated alternative splicing, a novel mammalian protein expression system, followed by SELEX-Seq, was used to identify a GU-rich RBM38 binding motif. Lastly, using a tethering assay, we determined that RBM38 can directly activate splicing when recruited to a downstream intron. Together, our data support the role of RBM38 in regulating alternative splicing during erythroid differentiation.

## Introduction

Alternative splicing of pre-mRNA transcripts generates protein isoforms with cell-type specific functions that are essential for development and homeostasis [1]. Splicing is regulated by the combinatorial control of RNA-binding proteins (RBPs). Ubiquitously expressed RNA-binding proteins such as the SR proteins and hnRNP proteins function in conjunction with tissue-specific splicing regulators, such as Nova 1/2, Fox proteins, and ESRP1/ESRP2 to combinatorially regulate alternative splicing [2-5]. Advances in high-throughput sequencing and bioinformatics have vastly increased our knowledge of alternatively spliced genes and pre-mRNA binding site positions of alternative splicing factors [6]{Irimia, and Blencowe, 2012 #12875}. RNA splicing maps have been generated using bioinformatics and biochemical approaches that often reveal a position-dependence of RNA splicing regulators binding in close proximity to alternatively spliced exons where the binding position can determine whether a given regulator promotes exon splicing or skipping [7].

We previously used a luciferase-based alternative splicing reporter to conduct a high-throughput cDNA expression screen for novel splicing regulatory proteins. This study uncovered two epithelial-specific alternative splicing factors, ESRP1 and ESRP2, that regulate Fibroblast Growth Factor Receptor 2 (FGFR2) splicing [5]. In addition to the ESRPs, this screen identified several other proteins that had not previously been shown to regulate splicing. Among those regulators was RBM38 (also known as RNPC1), which robustly altered splicing of the reporter, but was not required for regulation of endogenous FGFR2 splicing [5]. Further evidence supporting the role of RBM38 as an alternative splicing factor is provided by studies of SUP-12, an RBM38 ortholog found in *C. elegans*, that functions as an alternative splicing factor in worm muscle through binding to GU-rich motifs [8-10]. In humans, RBM38 has not been characterized as an alternative splicing factor, but has been shown to regulate transcript stability by binding the 3’-UTR of cell cycle regulators p21, p53, p63 and p73[11-18], along with stabilizing mRNA transcripts of the multifunctional RNA-binding protein, HuR[14], and the murine double minute-2 (MDM2) oncogene [12]. The above studies strongly implicated RBM38 as a versatile RNA-binding protein with various functions in different cell contexts. Given the presumed role of RBM38 in alternative splicing, we set out to identify its endogenous targets.

Using a splicing-sensitive microarray, we defined a set of RBM38-regulated exons and provided further evidence for a role of RBM38 as a regulator of alternative splicing. Given the high levels of RBM38 expression in cells of erythroid lineage, we subsequently examined RBM38 expression in erythroid differentiated CD34^+^ cells. We found that upregulation of RBM38 during late erythroid differentiation corresponds to splicing switches of RBM38-regulated transcripts. We also determined that upregulated RBM38 expression correlates with activation of EPB41 exon 16, a well-characterized alternative splicing event that occurs during late erythroid differentiation [19-25]. To characterize the mechanism of RBM38-regulated alternative splicing, we used Systematic Evolution of Ligands by Exponential Enrichment and high through-put sequencing (SELEX-Seq) to identify a GU-rich binding motif and lastly, we found that RBM38 activates splicing when directly tethered downstream of a cassette exon. Together, our findings reveal that RBM38 is a regulator of alternative splicing and strongly suggest that it contributes to an alternative splicing program switch that occurs during terminal erythroid differentiation.

## Materials and Methods

### Plasmids

Epb41 minigenes were a generous gift from John Conboy (Lawrence Berkeley National Laboratory, Berkeley, CA) [21]. Plasmids used to ectopically express 2X Flag-tagged Rbm38-FF and Rbfox2-FF proteins were described previously [5]. Plasmid pIPX-Rbm38-FF-TEV-SBP was constructed for mammalian protein expression. The SBP tag, which has been optimized for expression in mammalian cells, was PCR amplified from pIRESpuro-GLUE-hDsh3 [26] and subcloned into pINX-FF-TEV [5]. To generate a construct with a puromycin selectable marker, we subcloned FF-TEV-SBP from pINX-FF-TEV-SBP into pIPX [5]. Lambda N peptide and Box B constructs were a generous gift from Matthias Hentze (European Molecular Biology Laboratory, Heidelberg, Germany)[27]. For the lambda N-Box B tethering system, the 2xBox B sequence was PCR amplified with ClaI and XhoI sites and cloned into a previously described minigene pINE-PKC-40b-EGFP (A) or pIPL-PKC-40b-Luciferase(A) [28]. For Rbm38 fusion proteins, lambda N peptide was PCR amplified and cloned into the N- or C-terminus of a previously described protein expression vector pIBX-C-FF(B) [5] containing an NLS to generate constructs pIBX-N-λN-NLS-C-FF(B) or pIBX-C-FF(B)-NLS-λN. Gateway cloning strategies were used to clone the ORFs of a panel of RNA-binding proteins into pIBX-N-λN-NLS-C-FF(B). Nut R Box B nucleotide and λN amino acid sequences are 5’ UGGCCCUGAAAAAGGGCCA3’ and MNARTRRRERRAEKQAQWKAAN, respectively. Primer sequences are provided in Table S3. Sequences of all plasmids used are available upon request.

### Cell culture

MCF-7 cells were grown in DMEM-F12. RL-7 cells (ATCC) were grown in RPMI 1640. 293T cells were grown in DMEM. Cells were grown in medium supplemented with 10% FBS. Human primary cell culture was performed as previously described [29]. Erythroid and non-erythroid (G/M) differentiation of CD34^+^ umbilical cord blood was performed as previously described [29-31]. Flow cytometric analyses were performed for erythroid markers (CD45 FITC, GlyA PE, and CD71 APC) and GM markers (CD15 FITC, CD11b PE, and CD45 APC).

### Transfection

Transfection of MCF-7 cells with siRNA (Qiagen) (see supplement for sequence) was performed using Lipofectamine 2000, as previously described [5]. Transfection of RL-7 cells with siRNAs was performed by nucleofection using Amaxa kit V and T16 program. Co-transfections of 293T cells were performed as previously described [32].

### RT-PCR and qRT-PCR

For RBM38 knockdown experiments, RNA was collected 72 h post-transfection. Quantitative RT-PCR validations were performed as previously described [5,33]. Expression of RBM38 mRNA was analyzed using TaqMan assay (cat # Hs00766686_m1, Life Technologies).

### Immunoblotting

Cell lysates were collected 72 h post-transfection and were resolved on 4 to 12% NuPAGE Bis-Tris gels (Invitrogen) and immunoblotted as previously described [5]. RBM38 antibody was from Santa Cruz Biotechnology (SC-85873).

### Expression of fusion proteins for SELEX-Seq

Transient transfections of 293T cells using TransIT (Mirus Madison, WI) were performed in 10 cm cell culture plates following manufacturer’s recommendations. After 48 h, 1 mL of RIPA buffer was used to extract total protein from cells expressing fusion proteins. Total protein concentration was determined by Bradford analysis and the amount of fusion protein was determined by ECL plus analysis using pure Flag-tagged protein as a standard. We estimated that 1 mL RIPA extract contained 5 mg total protein and ~250 µg fusion protein.

### Purification and cleavage of fusion proteins

Fusion proteins were purified from cell lysates using Ultralink-immobilized streptavidin plus resin (Thermo Scientific) (binding capacity for SBP is 500 µg/mL packed resin [34]). 200 uL packed resin was washed three times at 4 °C for 5 min at 500xg using 400 L RIPA buffer supplemented with 50 mM NaCl to give a final concentration of 200 mM NaCl, 1.0% IGEPAL, 0.5% sodium deoxycholate, 0.1% SDS, and 50 mM Tris (pH 8.0) and supplemented with protease inhibitors. RIPA protein extracts containing ~100 µg fusion protein (as determined by ECL plus quantitation) were bound to resin by rotating at 4 °C for 4 h. Bound proteins were washed three times by rotating at 4 °C for 5 min followed by wash removal at 4 °C for 5 min at 500xg using 1 mL purification buffer: 16 mM HEPES (pH 7.9), 200 mM KCl, 2 mM MgCl_2_, 60 uM EDTA,1 mM DTT, 0.15 mM PMSF and 6% Glycerol. Purification buffer was exchanged three times, without rotation, using 1 mL of TEV cleavage buffer: 10 mM Tris (pH 8.0), 150 mM NaCl, 0.1% NP-40 0.5 mM EDTA, 1 mM DTT. The bound SBP tag was cleaved from Flag tagged protein using 20 µl of lab-generated recombinant TEV protease in 150 µl TEV cleavage buffer (equivalent to 100 U of 10 U/µl AcTEV protease), expressed from pRK793, according to a previously described protocol [35]. Fusion proteins were cleaved with TEV protease overnight at 4 °C with gentle rotation. The next day, resin with the TEV cleavage buffer was transferred to a 1 ml Micro Bio-Spin column (BioRad, Cat. # 32-6204) and centrifuged at 1,000xg for 5 min at 4 °C and ~150 µl eluate containing cleaved Flag-tagged protein was collected. An additional 100 µl TEV buffer was added to the spin column and a second elutate was collected. Concentrations of Flag-tagged proteins were determined by Coomassie stain analysis using a standard. 

### SELEX-Seq

RNA libraries were generated as previously described [36]. Briefly, 80 nt RNA libraries, containing a random 20-mer sequence, were transcribed from a 103 bp DNA pool using a MEGAscript T7 kit (Life Technologies). Proteins were prepared for SELEX by combining 2 µg Flag-tagged protein in 500 L 1x binding buffer (16 mM HEPES (pH 7.9), 150 mM KCl, 2 mM MgCl_2_, 60 µM EDTA, 1 mM DTT, 6% glycerol, and 0.15 mM PMSF) with 100 L anti-FLAG M2 affinity resin (Sigma A2220) (binding capacity for Flag is 600 µg/1 mL packed resin) by rotating at 4 °C for 30 min. A 20 µg randomized RNA pool was incubated with Rbm38-FF at a 11:1 molar ratio of RNA to protein in a 200 µL binding reaction mixture (16 mM HEPES (pH 7.9), 150 mM KCl, 2 mM MgCl_2_, 60 µM EDTA, 1 mM DTT, 6% glycerol, 0.01 µg/µL heparin [Sigma H-3393], 0.15 mM PMSF, and 0.2 U/µL SUPERase-In (Life Technologies). Binding reaction mixtures were incubated with rotation at room temperature for 30 min and washed three times with 1x binding buffer. Bound RNA was isolated from beads using TRIzol reagent according to the manufacturer’s instructions (Invitrogen). A cDNA library was prepared by random hexamer priming to isolated RNA, followed by reverse transcription using MMLV reverse transcriptase (Promega). This cDNA was then used as a template for T7 transcription to generate an RNA pool for the next round of SELEX or to prepare barcoded libraries for single lane high-throughput sequencing. This cycle was repeated for a total of three rounds. Sequencing libraries were prepared from round 0, 1, 2, and 3 cDNA pools using a PCR strategy with modified Illumina adaptor sequences containing four nucleotide barcodes specific to each round and sequenced with an Illumina HighSeq 2000 to generate 50-bp single-end reads [36]. Illumina sequence reads were analyzed as previously described [36]. 

### HJAY microarray

RNA purification, preparation of biotinylated sense-strand DNA,and hybridization was carried out as described previously [33]. The CEL file data sets can be accessed at NCBI GEO repository through accession number GSE49293. Data analysis was performed using the MADS+ computational pipeline to detect differential splicing events from the Affymetrix exon junction array data [37]. For each alternative splicing event, MADS+ evaluates the signals of probes targeting competing transcript isoforms to identify exons or splice sites with different levels of transcript inclusion between control and knockdown sample groups.

## Results

### Identification of RBM38 alternative spliced targets in MCF-7 breast cancer cells

**Figure 1:**
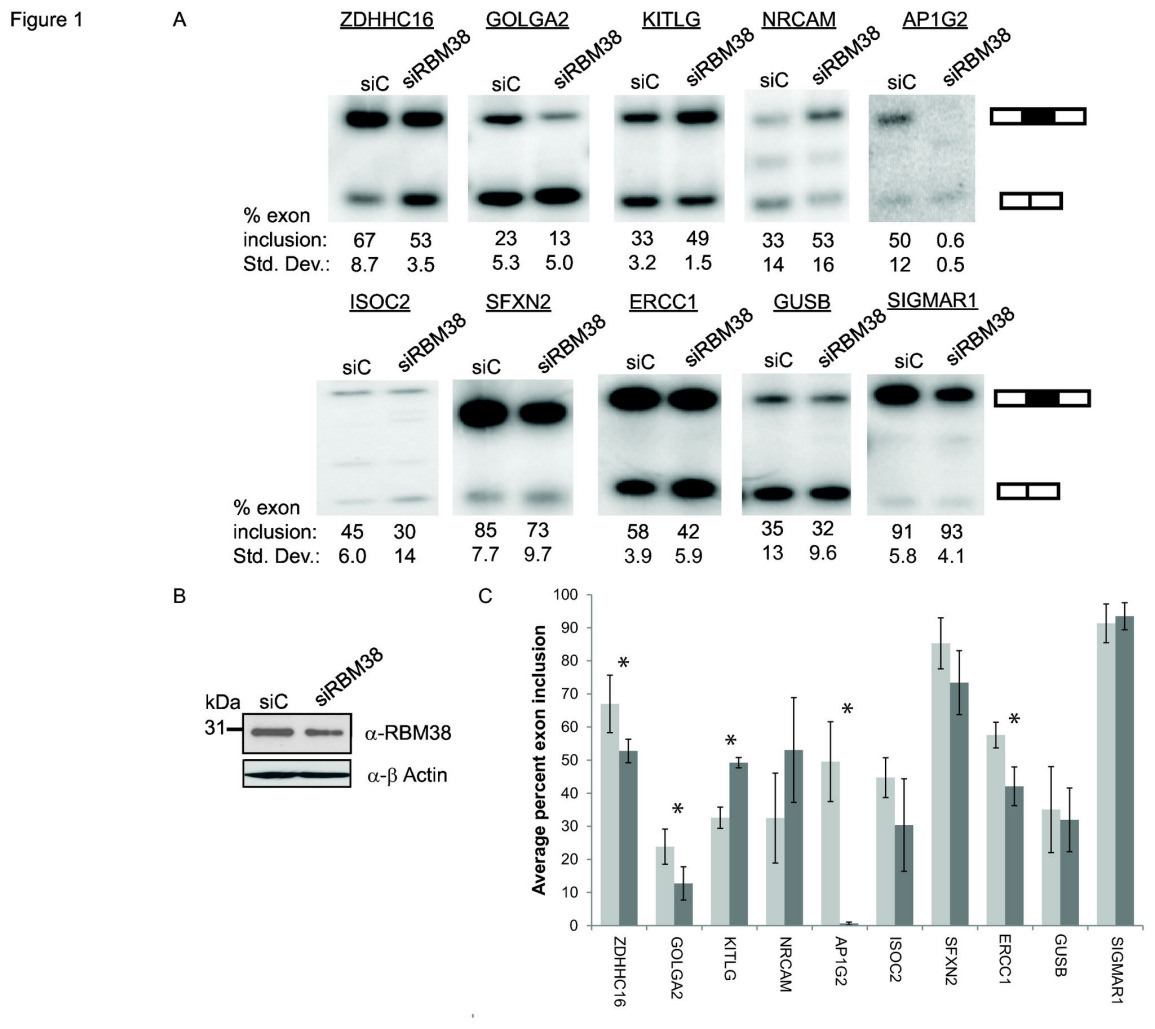
Validation of microarray predicted RBM38-regulated splicing events. A) Semi-quantitative RT-PCR analysis of HJAY microarray targets in MCF-7 cells. Average percentages of exon inclusion with standard deviations compiled from three experiments are indicated below a representative gel. . For AP1G2, only two replicates were included in analyses. B) Western blot detection of siRNA knockdown of RBM38 in MCF-7 cell line (upper panel). Actin is shown as a loading control (lower panel). C) Bar graph representing data from panel A. siControl and siRBM38 treated cells are shown in light and dark gray bars, respectively (*P*-values of ≤ 0.05 are annotated with an asterisk).

After identifying RBM38 as an activator of splicing in a high-throughput cDNA expression screen, we next investigated whether humans or other organisms express homologous RBM38 proteins that regulate splicing [5]. RBM38 and its mammalian paralog RBM24 have high sequence similarity, particularly within the single RNA Recognition Motif (RRM). In addition, a previously described ortholog of RBM38, SUP-12, from *C. elegans*, has been shown to function in tissue-specific muscle alternative splicing and displays high sequence similarity to RBM38 within its RRM (Figure S1). Given the association of RBM38 with alternative splicing, we next sought to identify its endogenous targets using cell types with high RBM38 expression (Figure S2) [38,39]. We noted relatively high levels of RBM38 expression in several cell lines, including MCF-7, a breast cancer cell line and RL-7, a blood cell line (Figure S2A). To identify alternatively spliced targets of RBM38, we first knocked down RBM38 in MCF-7 cells. RBM38-regulated splicing changes were detected using an Affymetrix human exon junction array (HJAY) and the MADS+ analysis pipeline, as previously described [32,37]. With a partial knockdown of RBM38, we identified 87 targets using previously described statistical thresholds, of which 8 out of 10 exons showed the same predicted direction of change in exon inclusion by semi-quantitative RT-PCR in 3 biological replicates (Figure 1, Figure S3A,B and Table S1). Of these, a paired Student’s t-test furthermore showed significant p-values for the predicted change in splicing (Figure 1C). However, GUSB and SIGMAR1 did not show the same change in splicing in three replicates and thus are not considered validated targets (Figure 1 and Figure S3).These data suggest that RBM38 can regulate splicing of endogenous target genes. However, the physiological relevance of these findings in a breast cancer cell line to RBM38 function *in vivo* is unknown, but could potentially be linked to cancer. For example, one RBM38-regulated splicing event, activation of excision repair cross complementation group-1 (ERCC1) exon 8, occurs in ovarian cancer cells and has been linked to cisplatin-resistance [40]. Nonetheless, to further investigate the *in vivo* functions of RBM38 regulated splicing we sought to identify potential roles of RBM38 in non-transformed cell types that more closely resemble cells where it is expressed *in vivo*. 

### RBM38 is highly expressed in hematopoietic cell types, particularly cells of the erythroid lineage

**Figure 2:**
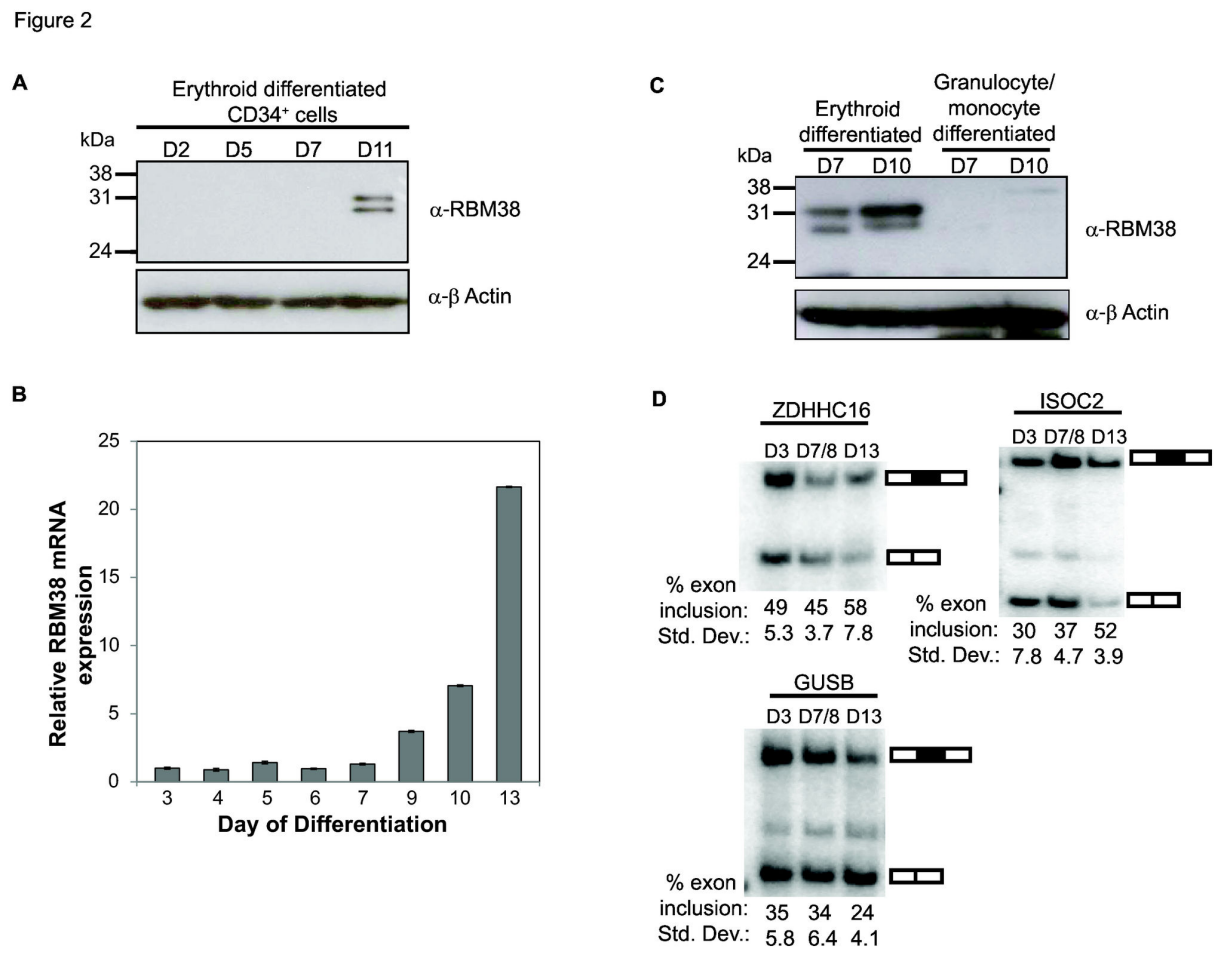
RBM38 is expressed during late erythroid differentiation and RT-PCR analysis of a subset of RBM38-regulated microarray targets in erythroid differentiated cells. A) Western blot detection of RBM38 in erythroid differentiated CD34^+^ cells on days 2, 5, 7, and 11. (upper panel). Actin is shown as a loading control (lower panel). B) Quantitative RT-PCR of RBM38 mRNA expression levels during erythroid differentiation on days 3 to 13. C) Western blot detection of RBM38 in erythroid or granulocyte/monocyte differentiated CD34^+^ cells on days 7 and 10. SuperSignal West Femto Chemiluminescent ECL reagent was used to detect lower levels of RBM38 in early erythroid cells. D) Semi-quantitative RT-PCR analysis of RBM38 microarray targets ZDHHC18, ISOC2, and GUSB in erythroid differentiated cells. Average percentages of exon inclusion and standard deviations from three experiments are indicated below a representative gel. For the middle time point, D7/8, average and standard deviation are calculated from one Day 7 and two Day 8 replicate samples.

We subsequently analyzed microarray data for gene expression programs in diverse human tissues and cell types and noted that RBM38 was predominantly expressed in hematopoietic cells, with some expression noted in muscle tissue [38,39]). We noted particularly high levels of RBM38 mRNA expression in CD71+ erythropoietic cells and based upon this evidence for expression in erythropoietic cells, we further examined the expression of RBM38 during erythropoiesis (Figure S2B). To assess the role of RBM38 during erythropoiesis, erythroid differentiation was induced using CD34^+^ hematopoietic stem cells purified from human umbilical cord blood, as previously described [29]. CD34^+^ cells propagate in culture and can be differentiated into mature hematopoietic cells of various lineages when cultured with lineage-specific cytokines [30,31]. We observed significant upregulated expression of RBM38 protein and mRNA in erythroid differentiated CD34^+^ cells (Figure 2A and 2B). To determine if RBM38 expression is specific to the erythroid lineage, we compared the level of RBM38 protein expression in erythroid and non-erythroid differentiated CD34^+^ cells (Figure 2C). We confirmed high levels of RBM38 protein expression in CD34^+^ cells induced along the erythroid lineage, but demonstrate that expression is absent in the non-erythroid (granulocytic/monocytic) lineage. These data suggest a specific role for RBM38 during erythropoiesis. 

### Analysis of alternative splicing switches in RBM38-regulated targets during red blood cell differentiation

 Given the upregulated expression of RBM38 during erythroid differentiation, we next tested nine RBM38 targets identified in MCF-7 cells to determine whether they switched splicing in a predicted manner (Figure 2D and Figure S3C,D). KITLG was not examined, as it is not expressed in terminally differentiated erythroid cells [41]. In the case of ZDHHC16 and ISOC2 we noted switches in splicing that were consistent with the change predicted based on increased RBM38 expression. However, for GUSB, the splicing switch was opposite to that predicted solely based on RBM38 mediated regulation. This was finding was not surprising, given that GUSB was not among the eight validated targets from the MCF-7 cell line. The remaining six exons did not reveal reproducible changes in splicing or could not be detected by RT-PCR (data not shown). We suspect that the differences in target gene splicing are partly due to the distinct transcriptome and proteome in these cell types. Changes in the expression of other splicing factors occur during erythroid development and hence it may be that for some RBM38 targets the combinatorial control of some of these exons by other regulators predominates over the effects of RBM38 alone.

### RBM38 regulates alternative splicing of erythroid specific EPB41 during red blood cell differentiation

**Figure 3:**
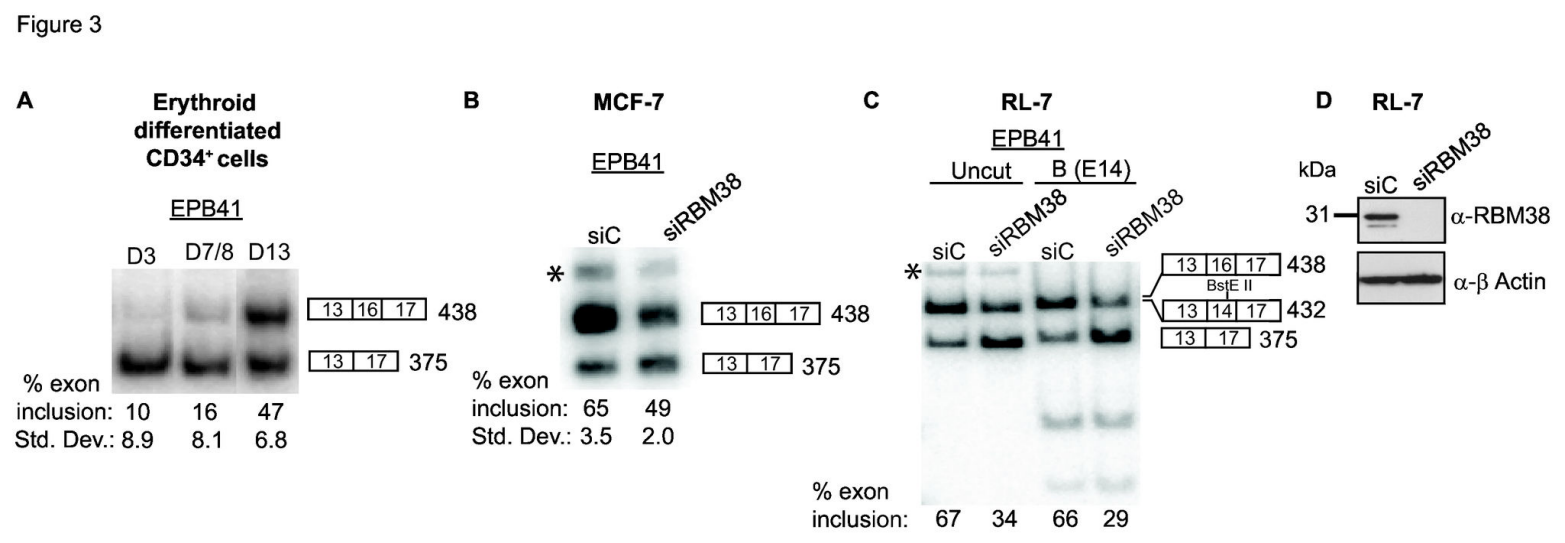
EPB41 exon 16 splicing increases during erythroid differentiation and exon 16 splicing is regulated by RBM38. A) Semi-quantiative RT-PCR detection of the activation of EPB41 exon 16 during late erythroid differentiation.).Average percentages of exon inclusion with standard deviations compiled from three experiments are indicated below a representative gel. For the middle time point, D7/8, average and standard deviation are calculated from one Day 7 and two Day 8 replicate samples. B) Semi-quantitative RT-PCR analysis of EPB41 exon 16 inclusion in MCF-7 cells after siRNA mediated knockdown of RBM38 from three biological replicate experiments. (C) RT-PCR analysis of EPB41 exon 16 splicing in response to knockdown of RBM38 in RL-7 cells RT-PCR products in RL-7 blood cell line were uncut or digested with BstE II (labeled as B) to detect presence of exon 14. D) Western blot detection of siRNA knockdown of RBM38 in RL-7 cells. Asterisk indicates a higher molecular weight RT-PCR product that is slightly enhanced in lanes with high RBM38 expression.

Previous studies have focused on the role of alternative splicing during erythropoiesis [19,42]. Perhaps the best example of alternative splicing during erythropoiesis involves EPB41 where a switch from exon 16 skipping to inclusion occurs from erythroid precursors to mature red blood cells. Inclusion of EPB41 exon 16 produces a protein isoform containing a cytoskeleton spectrin-actin binding domain, resulting in the necessary deformability characteristics of mature red blood cells [22-24,43]. Previous work has implicated Rbfox2 and hnRNPA1 proteins [20,21,44] in the multifactorial regulation of EPB41 splicing, yet there has also been evidence to support a role for other regulators. Given the corresponding upregulation in RBM38 expression during erythroid differentiation, we investigated whether RBM38 might play a significant role in activating splicing of EPB41 exon 16 during this process. Consistent with previous reports, we detected activation of Protein 4.1 (EPB41) exon 16 splicing during later stages of erythroid differentiation (Figure 3A) [19-21]. Unfortunately, we were unable to collect sufficient human erythroid cells to carry out RNAi directly in these cells. We therefore used siRNAs to knockdown RBM38 expression in the MCF-7 cell line, in which EPB41 was shown to be expressed. As expected, a partial knockdown of RBM38 resulted in increased skipping of EPB41 exon 16 (Figure 3B). We also selected the non-Hodgkin lymphoma B cell line, RL-7, as a hematopoietic cell line amenable to siRNA for analysis of EPB41 splicing in response to RBM38 knockdown. Similar to the MCF-7 cell line, we noted that knockdown of RBM38 in RL-7 cells also induced apparent EPB41 exon 16 skipping (Figure 3C and 3D). We observed an extra band in MCF-7 and RL-7 cells that might represent products containing exon 14 or 15 (both alone and/or together with exon 16). It should be noted that exons 14 and 15 are not included in red blood cells, but can be included in other cell types. Given that RL-7 cells are of hematopoietic origin and are most similar to the aforementioned primary erythroid cells, we chose to digest the RL-7 cell RT-PCR products with exon 14 specific BstEII or exon 15 specific HindIII. Digestion products were visible with BstEII, but not with HindIII (Figure 3C, right two lanes and data not shown). Thus, a small amount of EPB41 exon 14 is present in RL-7 cells, but did not significantly affect the calculated change in exon 16 percent exon inclusion. Using BstE II digested products, it was clear that a greater than 30% reduction in exon 16 splicing occurred in response to RBM38 knockdown. In addition, given the potential for the paralogous RBM24 to compensate for loss of RBM38 expression, we verified that RBM24 mRNA was not expressed in RL-7 cells, as well as CD34+ erythroid differentiated cells (Figure S4). 

### Rbm38 activates splicing in EPB41 minigenes

**Figure 4:**
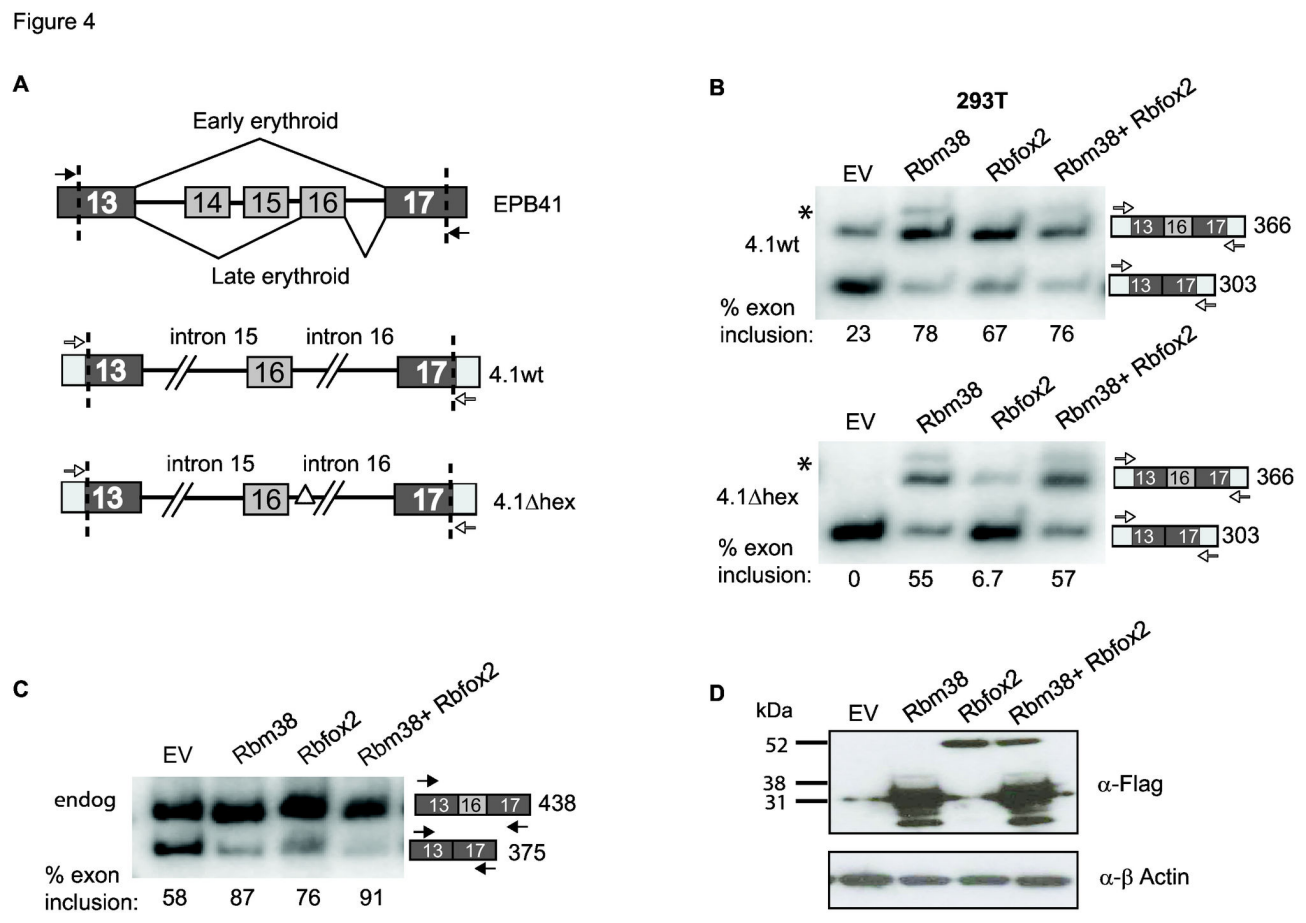
Rbm38 activates Exon 16 in Epb41 minigenes. A) Schematic depicting a portion of the mouse protein 4.1R (Epb41) gene from exons 13 to 17 (Upper panel). In early erythroid differentiation, exons 14-16 are skipped, and in late erythroid differentiation exon 16 is included. Dashed lines indicate the position of exon 13 and 17 truncations in the minigenes described below. Closed and open arrows indicate primer binding sites that specifically amplify mRNA expressed from the minigenes and endogenous Epb41 locus, respectively. 4.1wt is a 1.2 kb minigene with indicated exonic and intronic sequences (Middle panel). Minigene 4.1Δhex lacks all three UGCAUG Rbfox2 binding motifs due to a 186-nt deletion within intron 16 (Lower panel). B) Co-transfection of minigenes and Empty vector (EV), Rbm38-FF, Rbfox2-FF, or both Rbm38-FF and Rbfox2-FF. *Upper* *panel*, expression of Rbm38 and Rbfox2 promotes inclusion of Exon 16 in the 4.1wt minigene. When Rbm38 and Rbfox2 are expressed together, there is no further enhancement of exon 16 inclusion. *Lower* *panel*, expression of Rbm38 promotes inclusion of Exon 16 in the 4.1 Δhex, while Rbfox2 has little effect. When Rbm38 and Rbfox2 are expressed together, activation of exon 16 splicing is similar to Rbm38 alone. We also noted a small band above the exon 16 inclusion product in Rbm38 lanes (Figure 3B, asterisk). This band was sequenced and found to contain exon 13, a retained intronic sequence downstream of exon 13, a region from intron 15, and exon 17. Exon 16 was not included. C) RT-PCR of endogenous EPB41 exon 16 inclusion in response to transfection of EV, Rbm38, Rbfox2, or both Rbm38 and Rbfox2. Percent exon inclusion is indicated below each lane. RT-PCR product sizes are provided to aid the reader in distinguishing minigene Epb41 from endogenous EPB41. D) Western blot analysis of Rbm38 and Rbfox2 protein expression levels. Actin was used as a loading control.

 Our finding that knockdown of RBM38 induced skipping of EPB41 exon 16 strongly implicates it as a regulator of this splicing switch during erythroid differentiation, but it is possible that this effect could be indirect. For example, previous studies showed that RBM38 can influence mRNA stability through binding to 3’ UTRs [11,16-18] and hence it might modify the expression of other splicing regulators to induce these splicing changes. As a first step to test whether this regulation is direct we performed co-transfection assays in 293T cells with two previously described mouse Epb41 minigenes, 4.1wt and 4.1Δhex. Minigene 4.1wt contains Epb41 exons 13, 16 and 17 and portions of flanking intronic sequences, while minigene 4.1Δhex has a region of intronic sequence deleted downstream of exon 13 that includes three Rbfox2 binding site motifs, UGCAUG (Figure 4A). Previous work by Conboy and colleagues showed that Rbfox2 could induce exon 16 splicing in the 4.1wt minigene, but not when the consensus Rbfox2 binding sites were deleted in the 4.1Δhex minigene [21]. Consistent with their report, Rbfox2 activated splicing of exon 16 when co-transfected with the 4.1wt minigene, but had a minimal effect on splicing when co-transfected with minigene 4.1Δhex (Figure 4A and B, lane 3). In contrast, RBM38 robustly enhanced exon 16 splicing in both minigenes, indicating that neither the Rbfox2 binding motif nor other deleted intron sequences are required for splicing activation (Figure 4A and B, lane 2). 

Using the same RNA samples, we also examined the effect of ectopic expression of Rbm38, Rbfox2, or both proteins together on alternative splicing of endogenous EPB41 exon 16 (Figure 4C). Rbm38 and Rbfox2 both individually activated splicing of endogenous EPB41 exon 16, and together, Rbm38 and Rbfox2 activated slightly greater than when expressed alone, although the same additive effect was not observed in the co-transfected minigenes. Western blot analysis of Flag tagged protein expression is provided in 4D. Together, these data suggest that Rbm38 regulates activation of EPB41 exon 16 in an Rbfox2 independent manner and that it does not require the binding sites used by Rbfox2 to regulate the same exon.

### SELEX-Seq analysis reveals a GU-rich Rbm38 binding motif

 In order to further characterize mechanisms by which RBM38 regulates splicing, we used Systematic Evolution of Ligands by Exponential Enrichment (SELEX) coupled with high-throughput sequencing (SELEX-Seq) to define the RBM38 binding motif. Our lab previously used this approach using a recombinant GST-Esrp1 fusion protein generated in *E. coli* [36]. Given that many mammalian proteins can be difficult to express in bacteria, we developed a modified approach to express sufficient quantities of Rbm38 for binding studies in 293T cells, similar to an approach previously used for SELEX with transcription factors [45]. We developed an expression construct in which a cDNA of interest (e.g. Rbm38) can be expressed with a C-terminal tandem affinity tag containing two FLAG tags and a Streptavidin Binding Peptide (SBP) tag, and separated by a Tobacco Etch Virus (TEV) protease cleavage site (FF-SBP tag). The streptavidin binding peptide tag is particularly advantageous given its tight nanomolar binding affinity for streptavidin [34] and the convenience of such a tag for use in various applications requiring streptavidin conjugated resins, plates, or beads. 

**Figure 5:**
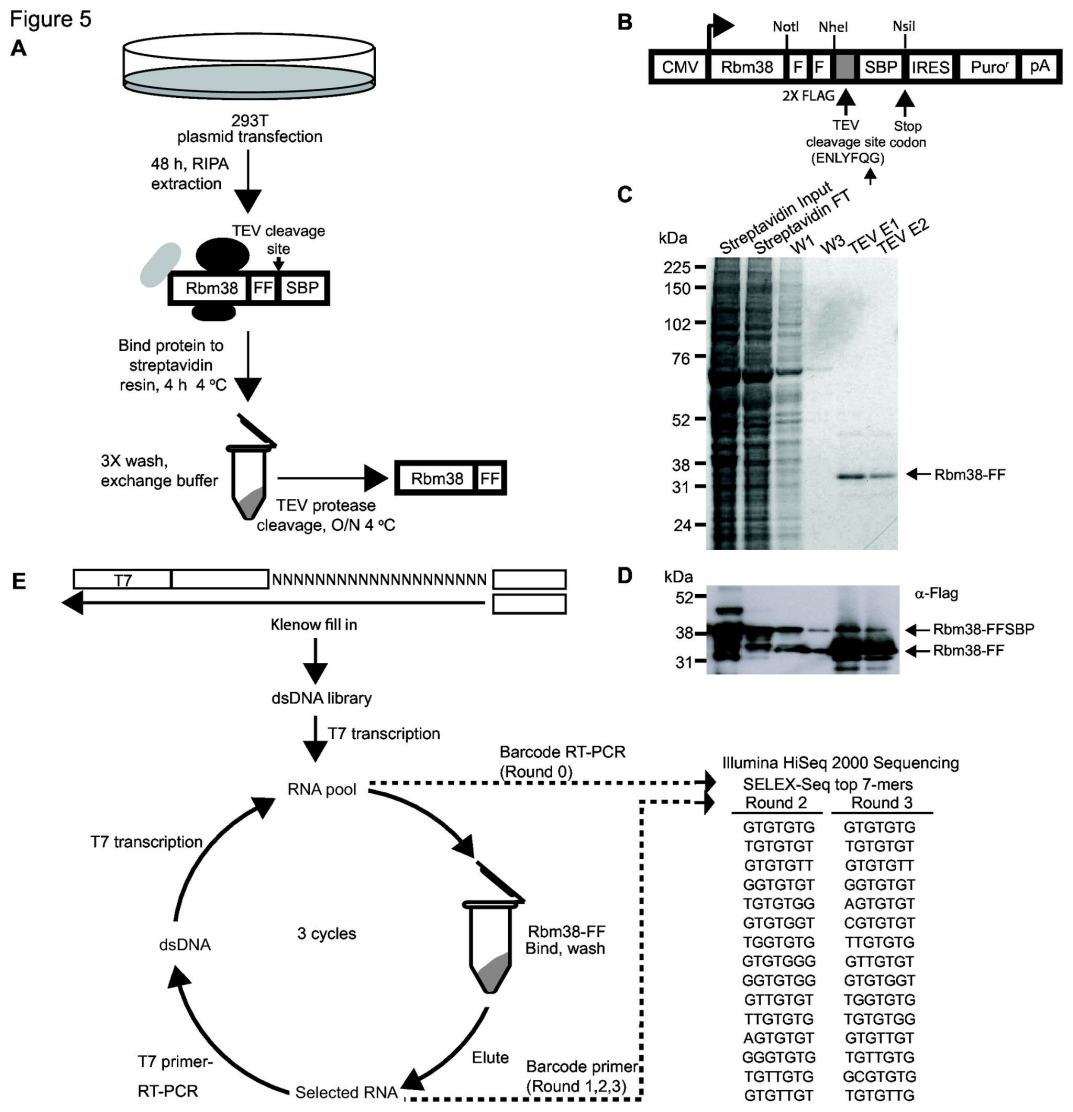
Mammalian expression and purification of Rbm38, followed by SELEX-Seq analysis to identify an RBM38 binding motif. A) Schematic of mammalian expressed Rbm38-FF-SBP purification steps. Mammalian 293T cells were transiently transfected for 48 h and protein was extracted using RIPA buffer. RIPA extract was added to Streptavidin resin and washed as indicated. The SBP tag was cleaved by incubation with TEV protease. Purified Rbm38-FF was used for SELEX-Seq. B) Map of FF-SPB tagged mammalian protein expression vector used in this study. Coomassie stain (C) and western blot (D) analysis of samples collected during purification of Rbm38-FF-SBP. E) Schematic for SELEX-Seq protocol. SELEX-Seq 7-mer motifs identified after two and three rounds of selection.

We transiently transfected mammalian 293T cells with a cDNA for Rbm38 containing the FF-SBP tag and collected total cell extract for protein purification (Figure 5A-D). After immobilization on streptavidin resin, washes with moderately high salt, and cleavage of FLAG-tagged Rbm38 from the resin with TEV protease we noted that the RBM38 protein purified by this method was relatively pure with minimal to absent co-purifying proteins (Figure 5C, lane TEV E1). We also monitored Flag tagged Rbm38 during purification and TEV cleavage by western blot (Figure 5D). We noted that most of the SBP tag was efficiently cleaved by Tobacco Etch Virus (TEV) protease. As a positive control for SELEX-Seq, Esrp1-FF-SBP was purified in parallel with Rbm38-FF-SBP. Purity of TEV cleaved Esrp1-FF-SBP was similar to that of Rbm38-FF-SBP (data not shown). Given the success in obtaining sufficient quantities of pure protein from the 293T expression system we proceeded to carry out SELEX-Seq using an *in vitro* transcribed RNA library containing a random 20-mer sequence and carried out three rounds of selection (Figure 5E). Barcoded sequencing adapters were added to each library for single lane 50 bp SR Illumina HiSeq 2000 sequencing. Using SELEX-Seq, we identified a GU-rich Rbm38 binding motif by the second round of selection (Figure 5E and Table S2). While some reports have suggested a GU-rich RBM38 binding motif, others have put forth variants of this motif. For example, alternative splicing factor SUP-12, the RBM38 ortholog from *C. elegans*, has been shown to bind GU-rich motifs [8,9]. In contrast, RBM38 has been shown to stabilize mRNA transcripts by binding AU, CU or U-rich motifs located in the 3’ UTR of p63, MDM2, HuR [12,16,17]. In addition, data obtained using individual-nucleotide resolution Cross-Linking and ImmunoPrecipitation (iCLIP) suggested that RBM38 binds a U-rich motif [46]. To ensure that the identified Rbm38 GU-rich binding motif is accurate, we performed SELEX-Seq of mammalian purified Esrp1 in parallel with Rbm38. The results using Esrp1 are in agreement with the previously published UGG-rich motif, which validated our method (data not shown) [36]. Further evidence that the SELEX-derived motif represents the optimal RBM38 binding site is provided by a recent study from Hughes and colleagues that used the RNAcompete method to derive an RBM38 binding site [47]. Of note, the top 7-mer motif from their study exactly matches that derived by SELEX-Seq, further supporting this as a direct binding site, at least based upon *in vitro* analysis. In addition, our success using this method to purify proteins expressed in human cells illustrates the nearly unlimited potential for this approach to characterize larger numbers of binding sites for numerous RNA binding proteins without the need for *E. coli* or baculovirus-based expression systems.

### Tethering studies with Rbm38 downstream of an alternative exon demonstrate that it can directly regulate splicing

Based upon the results from SELEX-seq, we suspected that GU-repeat type elements would mediate binding and regulation of splicing by RBM38 for EPB41 as well as other target transcripts. We therefore searched for conserved GU-rich sequence motifs within these regulated exons as well as in flanking intronic sequences where splicing factors typically bind regulated target exons. We identified two conserved GU-rich sequences in the intron downstream of EPB41 exon 16 that were retained in both the 4.1wt and 4.1Δhex minigenes. We thus made three additional minigene constructs with deletions at one or both GU-rich sites in the background of 4.1wt. However, deletion of individual or both of these elements did not significantly impair the ability of Rbm38 to activate splicing of exon 16 when co-transfected with these deletion minigenes (data not shown). Although the minigene studies strongly suggest that Rbm38 directly regulates Epb41, we were unable to further confirm this using deletion of candidate RBM38 binding sites. It is possible that other more degenerate, non-GU type elements, including those described in 3’ UTRs mediate direct binding. Hence a more exhaustive and systematic analysis to define these binding sites in EPB41 and other regulated transcripts may be needed to uncover these sites if indeed the regulation is direct. However, we cannot rule out the possibility that Rbm38 indirectly regulates transcript splicing. For example, given the evidence that RBM38 regulates mRNA stability [11-18], it is possible that these changes in splicing could be due to changes in expression of other alternative splicing regulatory factors.

**Figure 6:**
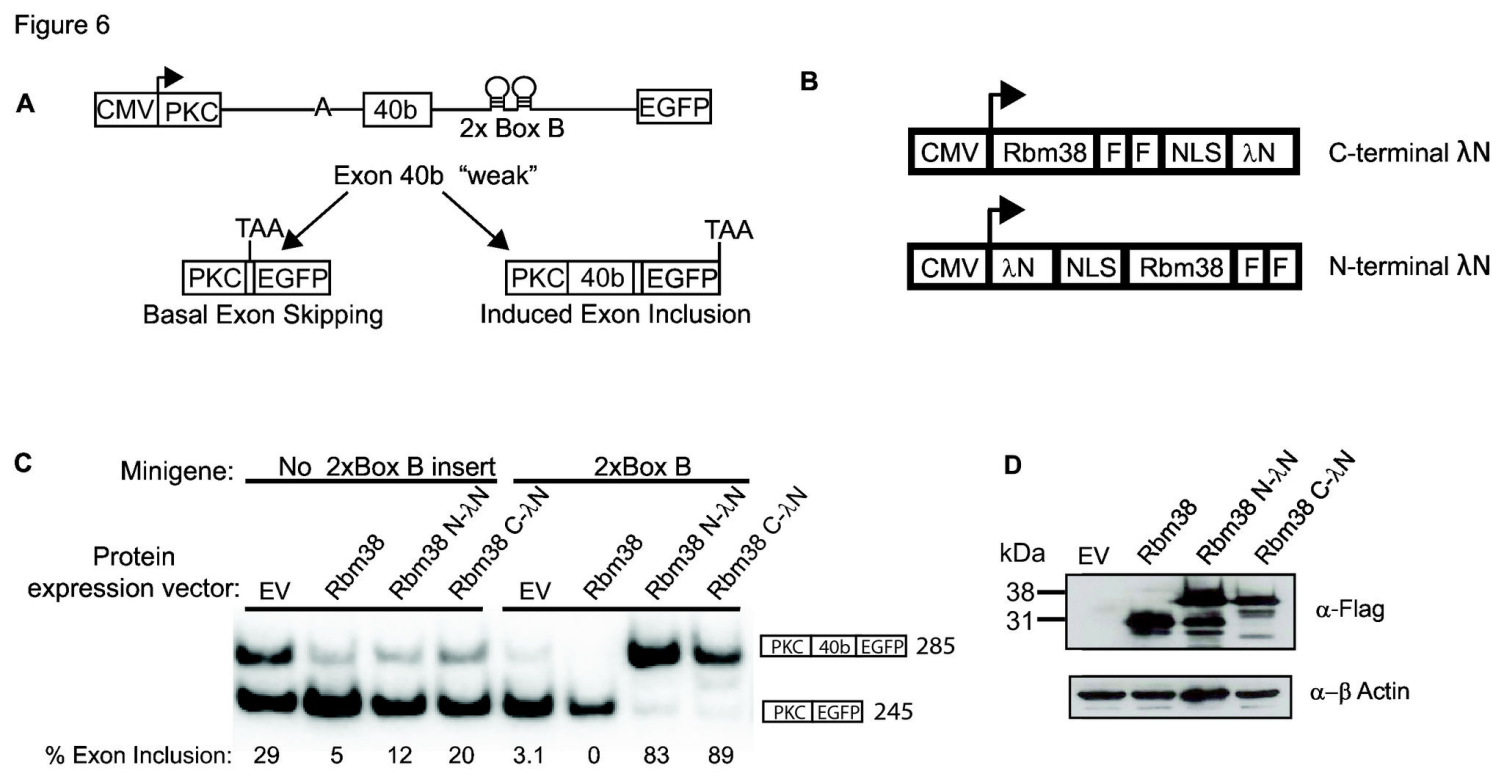
Direct tethering of Rbm38 to an intronic position downstream of a regulated exon activates splicing. A) Schematic of the PKC-40b-2xBoxB FGFR2 reporter minigene used in the lambda N-Box B tethering system. The minigene has a weak splice site, which promotes basal 40b exon skipping. The RNA sequence of Nut R Box B is provided in Materials and Methods B) Map of C- and N-terminal λN protein expression vectors. The amino acid sequence of lambda N peptide is provided in Materials and Methods C) Activation of 40b exon was examined by RT-PCR using RNA extracted from 293T cells transiently co-transfected for 48 h with expression vector: empty vector control (EV), Rbm38-FF, Rbm38-FF N-λN, or Rbm38-FF C-λN and minigene: no Box B insert or 2x Box B. Percent exon inclusion is provided below each lane. D) Western blot analysis of Flag tagged Rbm38 proteins used in (C).

 A number of splicing factors have been shown to preferentially promote exon splicing when bound to sequences in the downstream intron [48,49]. In order to further investigate whether Rbm38 can directly regulate splicing, we tethered Rbm38 to a downstream intron, using a lambda N-Box B tethering system [27,50]. The high nanomolar affinity between bacteriophage lambda N peptide and the Box B RNA hairpin loop makes this a convenient system for use to investigate RNA position dependence of RNA binding proteins. Using a previously described minigene [28], we positioned two Box B hairpins in the downstream intron of an artificial alternatively spliced exon 40b. Baseline inclusion of exon 40b is low, while positioning of alternative splicing factor binding motifs at the same approximate position in the downstream intron has been shown to enhance activation of exon 40b (Figure 6A, data not shown)[5,28]. In this study, we examined Rbm38 regulation of two minigenes, one with no Box B elements and the other with 2x Box B RNA hairpin elements. To ensure Rbm38 activity is not affected by the lambda N peptide, we prepared two fusion proteins with lambda N positioned on either the N- or C- terminus of Rbm38 (Figure 6B). First, we co-transfected the minigene without Box B elements along with a control empty protein expression vector (EV), Rbm38, Rbm38 N-lambda N, or Rbm38 C-lambda N (Figure 6C, left panel). In the absence of a Box B hairpin element, we expected low baseline inclusion of exon 40b and no change in splicing in response Rbm38 or Rbm38-lambda N expression. However, baseline inclusion was slightly elevated and Rbm38 expression silenced exon 40b. While Rbm38 or lambda N binding sites have not been engineered into this vector, it is possible that Rbm38 is able to repress splicing through combinatorial control with other splicing factors. Next, we co-transfected the 2x Box B minigene along with an EV control, Rbm38, Rbm38 N-lambda N, or Rbm38 C-lambda N (Figure 6C, right panel). In this case, baseline inclusion was low and, as predicted, co-expression with Rbm38 alone had no effect and Rbm38-lambda N fusion proteins induced strong activation of splicing. It is possible that the splicing activation observed in these minigenes could be indirect, for example by disrupting binding of proteins that otherwise silence splicing of the test exon. Therefore, we also tested the effects of recruiting a panel of known splicing regulators, such as RBFOX2, which activates splicing when bound downstream of a regulated exon, as well as other splicing regulators such as hnRNPC and hnRNPLL that are less predicted to activate splicing when bound to a downstream intron.These studies showed that known splicing factor, RBFOX2, was also promote splicing when recruited to the Box B elements, whereas hnRNP C and hnRNP LL do not (Figure S5A and S5B). In addition, the recruitment of a control protein without RNA-binding motifs, USP48, also did not affect splicing (Figure S5B). Together, these data provide evidence that the RBM38 protein can function to directly regulate exon splicing when bound to a regulated transcript. However, these data strongly imply, but do not prove that the regulation of the endogenous targets described previously is in fact direct. 

## Discussion

In this report, we present evidence supporting the role of RBM38 as a regulator of alternatively spliced transcripts during erythroid terminal differentiation. The inclusion of EPB41 exon 16, which produces a protein isoform that is necessary for the malleability of red blood cells, is the best characterized alternative splicing event during erythroid differentiation. While previous reports have implicated Rbfox2 and hnRNPA1 as important regulators of alternative splicing of EPB41 exon 16 during erythropoiesis [20,21,44], we provide evidence supporting the role of RBM38 as another regulator of this event. Erythroid differentiation of human CD34+ cells showed an upregulation of RBM38 expression that occurred in parallel with the activation of EPB41 exon 16. We also noted that several other putative RBM38 target exons identified in a separate microarray-based analysis that were also regulated during erythroid development. 

Rbfox2 has been implicated in the activation of Epb41 exon 16 by binding UGCAUG-rich intronic regulatory sites downstream of Epb41 exon 16 [20,21]. We characterized Rbm38 regulation of Epb41 using the same minigenes that have been used to investigate Rbfox2 regulation of Ebp41 [21]. We show that expression of Rbm38, as well as Rbfox2, promote robust activation of exon 16 splicing. Deletion of the Rbfox2 binding sites mostly abrogates splicing activity, while Rbm38 retained its ability to activate splicing in the absence of the Rbfox2 binding sites. It is therefore evident that while Rbm38 and Rbfox2 may cooperatively promote exon 16 splicing, they do so via interactions with distinct sequence motifs or alternative mechanisms. 

**Figure 7:**
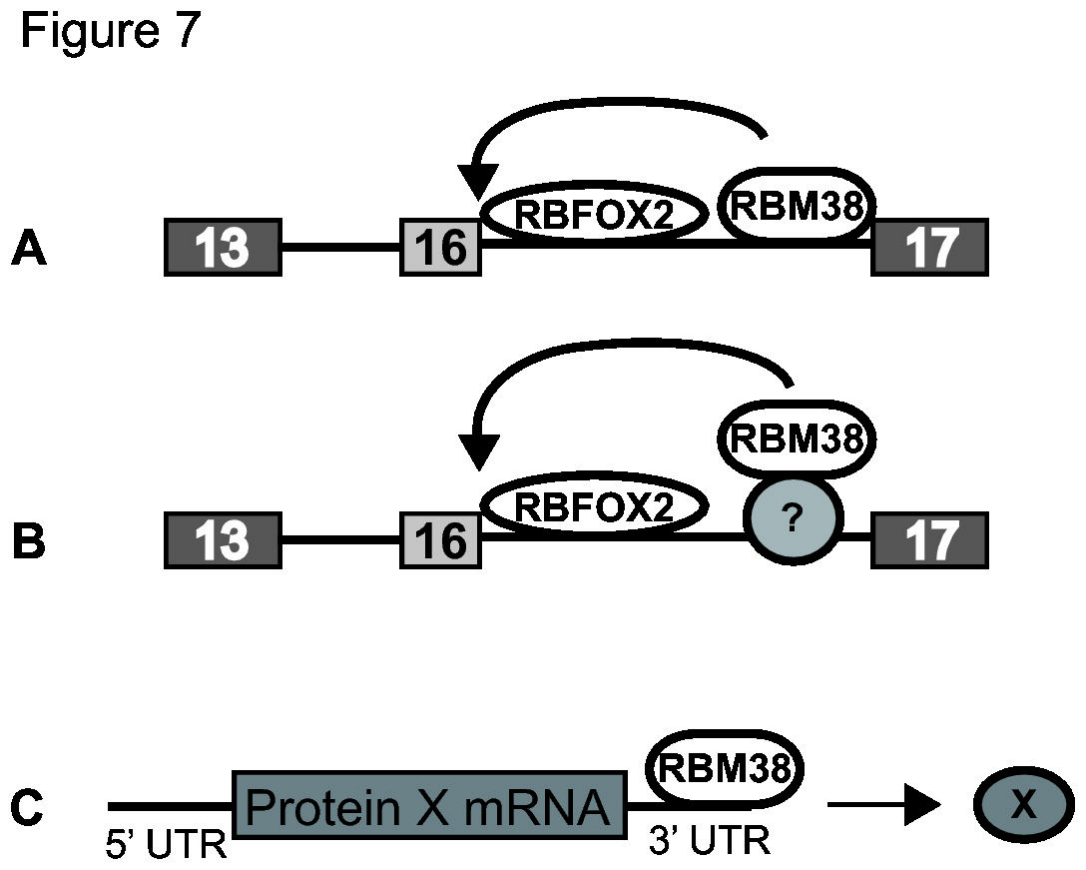
Models for RBM38-regulated activation of EPB41 exon 16. Our tethering data supports the ability of RBM38 to activate splicing by localizing downstream of a cassette exon. A) RBM38 may regulate alternative splicing by directly binding downstream of a regulated exon. B) RBM38 and other unknown RNA binding proteins may activate splicing through combinatorial control. C) RBM38 is known to bind the 3’UTR of mRNA transcripts and thus may stabilize transcripts and effect translation of unknown proteins, such as splicing factors.

To provide further mechanistic characterization of Rbm38 regulation of alternative splicing, we used SELEX-Seq to determine a GU repeat-type binding motif for Rbm38 that is likely to represent an optimal cis-element for RBM38 mediated splicing activity. As noted, a similar GU-rich element was previously shown to be bound by the *C. elegans* orthog SUP-12, providing further support for this motif [9,10]. However, when we deleted two short GU-rich regions downstream of Exon 16 in the 4.1Δhex minigene, we did not impair the ability of RBM38 to promote splicing of EPB41 exon 16. We were similarly unable to validate GU-rich motifs in other RBM38 target exons as mediating direct regulation of splicing (data not shown). While we cannot rule out the possibility that RBM38 binds AU, CU, or U-rich or AU rich elements [12,16,17], it is notable that these previous studies did not perform detailed analysis to confirm binding to specific motifs within the proposed 3’ UTR target regions. However, it is also possible that true *in vivo* binding sites may be more degenerate than the types of high affinity binding sites that typically emerge from SELEX. We are therefore presently unable to provide irrefutable evidence that RBM38 directly targets these transcripts’ splicing. It is thus possible that the effects on splicing we observe are indirect. However, the short time course of the splicing activation in the minigene assays is more consistent with direct regulation. Furthermore, experiments in which Rbm38 can directly activate splicing when tethered downstream of a cassette exon supports a direct role of Rbm38 in regulating splicing. It is therefore possible that there are either shorter GU- or U-rich sequence elements that mediate direct regulation. Alternatively, it is also possible that while RBM38 can directly bind to RNA, it may in certain contexts do so in cooperation with another regulator that mediates the protein-RNA interactions. Based on our data, we propose three models of RBM38-regulated activation of splicing (Figure 7). In the first model, RBM38 directly binds an undefined intronic enhancer element (ISE) and is able to activate splicing of EPB41 exon 16. In the second model, RBM38 associates with an unknown alternative splicing factor that directly binds the pre-mRNA transcript near the regulated exon and thereby influences exon splicing. A third model would be that RBM38 binds to the 3’ UTR and regulates mRNA stability or translation of other splicing regulators or transcription factors that control their expression to indirectly alter splicing. Although our data provide less support for the latter model, it is a still a possibility given previous reports supporting the role of RBM38 in binding and stabilizing the 3’-UTR of cell cycle regulators. 

 In summary, we identified Rbm38-regulated alternatively spliced gene transcripts, including erythroid specific EPB41 inclusion of exon 16. The correlation of RBM38 expression and a switch in RBM38-regulated isoforms during terminal erythroid differentiation suggests these RBM38 plays a significant role in splicing changes during this process. In addition to its role in alternative splicing, RBM38 has been implicated as a versatile protein with multiple functions to influence transcript fate, including stabilization of transcripts by binding to the 3’ UTR and miRNA protection [46], and thus further studies are warranted in order to reveal the full extent of RBM38 mediated alternative splicing and its other post-transcriptional functions in different cell contexts. 

## Supporting Information

Figure S1 **Sequence conservation of RBM38.** Clustal W2 (DNASTAR) alignment of the vertebrate RBM38 and RBM24, as well as the RBM38 *C. elegans* ortholog SUP-12. Conserved or similar amino acids are indicated with a star or colon, respectively. RRM motifs RNP1 and RNP2 are shown in blue. Non-conserved amino acids within the RRM are shown in red. The RRM domain, shown as black bar, is defined using Swiss-Prot (http://ca.expasy.org/sprot/).
(DOCX)

Figure S2 **Expression of RBM38 across a panel of cell lines and cell types.** A) Gene expression of RBM38 in NCI60 cell lines [39]. B) Gene expression of RBM38 in GeneAtlas U133A affymetrix microarray [38]. Cells lines or cell types that were examined or discussed in the text are denoted with an asterisk.
(EPS)

Figure S3 **Validation of microarray predicted RBM38-regulated splicing events.** A and B) Semi-quantitative RT-PCR analysis of HJAY microarray targets in MCF-7 cells from two additional biological replicate experiments. Percent exon inclusions are provided and were used to calculate average percentages of exon inclusion and standard deviations in Figure 1. C and D) Semiquantitative RT-PCR from additional two additional replicates from the experiments shown in Figure 2D.
(EPS)

Figure S4 **RT-PCR analysis of RBM24 expression.** Expression of RBM24 in (A) RL-7 cell line and (B) erythroid differentiated CD34^+^ cells. BT549 and T47D are shown as positive controls for RBM24 expression. RT-PCR for actin is shown as a loading control.
(EPS)

Figure S5 **Direct tethering of alternative splicing factors to an intronic position downstream of a regulated exon activates splicing.** A) Schematic of the PKC-40b-2xBoxB-Luciferase FGFR2 reporter minigene used in the lambda N-Box B tethering system. B) Activation of 40b exon was examined by RT-PCR using RNA extracted from 293T cells transiently co-transfected for 48 h with expression vector: empty vector control (EV), hnRNP C C-λN, hnRNP F C-λN, hnRNP LL C-λN, RBFOX2-FF C-λN, RBM24-FF C-λN, RBM39-FF C-λN, RBM38-FF C-λN or USP48 C-λN and 2x Box B minigene. Percent exon inclusion is provided below each lane (*upper panel*). Western blot analysis of Flag tagged proteins used in tethering assay (*lower panel*).
(EPS)

Table S1 **Complete set of results from HJAY analysis in MCF-7 cells with RBM38 knockdown by siRNA.** Column headings include hg19 chromosomal coordinates, Affymetrix assigned transcript IDs, junction probeset, exon probeset IDs, as well as specific exons IDs. Following each probeset, a splicing index direction of change in signal is shown with “-“ or “+” and indicates the junction change in response to RBM38 knockdown. The predicted effect of RBM38 expression is indicated with “enhance” or “silence”. Targets are ranked by the lowest p-value among the probsesets that predict the change in splicing. Targets examined in this study are shown in red.
(XLSX)

Table S2 **Rbm38 binding motifs identified from SELEX-Seq analysis.** List of 6-mer and 7-mer Rbm38 binding motifs for rounds 0 to 3 of SELEX. Read count, total read number, and fraction of total reads is provided.
(XLSX)

Table S3 **List of primer and oligonucleotide sequences used in this study.**
(DOCX)
