# Supplementary material for: The RNA Binding Protein RBM38 (RNPC1) Regulates Splicing during Late Erythroid Differentiation
Source: PLoS One. 2013 Oct 18;8(10):e78031. doi: 10.1371/journal.pone.0078031 (PMC3820963; doi:10.1371/journal.pone.0078031)
Supplement: Figure S1 — Sequence conservation of RBM38. Clustal W2 (DNASTAR) alignment of the vertebrate RBM38 and RBM24, as well as the RBM38 C. elegans ortholog SUP-12. Conserved or similar amino acids are indicated with a star or colon, respectively. RRM motifs RNP1 and RNP2 are shown in blue. Non-conserved amino acids within the RRM are shown in red. The RRM domain, shown as black bar, is defined using Swiss-Prot (http://ca.expasy.org/sprot/). (DOCX) [file pone.0078031.s001.docx]

**Figure S1**

H.sapiens_RBM38 --MLLQPAPCAPSAGFPRPLAAPGAMHGSQKDTTFTKIFVGGLPYHTTDASLRKYFEGFG 58

M.musculus_Rbm38 --MLLQPA-CSPSV-FPRPSAAPSAMHGSQKDTTFTKIFVGGLPYHTTDASLRKYFEGFG 56

D.rerio_rbm38 -------------------------MHTTQKDTTYTKIFVGGLPYHTTDSSLRKYFEVFG 35

H.sapiens_RBM24 -------------------------MHTTQKDTTYTKIFVGGLPYHTTDASLRKYFEVFG 35

C.elegans_SUP-12 MYGQVQDPLVHAAAAALAQSTNAEPVVGS-RDTMFTKIFVGGLPYHTSDKTLHEYFEQFG 59

: : :** :************:* :*::*** **

H.sapiens_RBM38 DIEEAVVITDRQTGKSRGYGFVTMADRAAAERACKDPNPIIDGRKANVNLAYLGAKPRSL 118

M.musculus_Rbm38 DIEEAVVITDRQTGKSRGYGFVTMADRAAADRACKDPNPIIDGRKANVNLAYLGAKPRSL 116

D.rerio_rbm38 EIEEAVVITDRQTGKSRGYGFVTMADRSAADRACKDPNPIIDGRKANVNLAYLGAKPRVM 95

H.sapiens_RBM24 EIEEAVVITDRQTGKSRGYGFVTMADRAAAERACKDPNPIIDGRKANVNLAYLGAKPRIM 95

C.elegans_SUP-12 DIEEAVVITDRNTQKSRGYGFVTMKDRASAERACKDPNPIIDGRKANVNLAYLGAKPRTN 119

:**********:* ********** **::*:***************************

H.sapiens_RBM38 QTGFAIGVQQLHPTLIQRTYGLTPHYIYPPAIVQPSVVIP----AAPVPSLSSPYIEYTP 174

M.musculus_Rbm38 QTGFAVGVQQLHPTLIQRTYGLTPHYIYPPAIVQPSVVIP----ATPVPSLSSPYLEYTP 172

D.rerio_rbm38 QPGFTFGVPQIHPAFIQRPYGIPTHYVYPQAFMQPSVVIPHIQPTATSATASSPYIDYTG 155

H.sapiens_RBM24 QPGFAFGVQQLHPALIQRPFGIPAHYVYPQAFVQPGVVIPHVQPTAAAASTT-PYIDYTG 154

C.elegans_SUP-12 VQLAALAAGQVQLPLTTQ----------LQALFQP------------------------- 144

:... *:: .: : *:.**

H.sapiens_RBM38 ASPAYAQY--------PPATYDQYPYAASPATAASFVGYSYPAAVPQALSAAAPA----- 221

M.musculus_Rbm38 ASPAYAQY--------PPATYDQYPYAASPAAATSFVGYGYPAAVPQALSAAAPA----- 219

D.rerio_rbm38 A--AYAQY-ASAATAAAAAAYEQYPYAASPAATGYVAAAGYGYAMQQPLATAAPG----- 207

H.sapiens_RBM24 A--AYAQYSAAAAAAAAAAAYDQYPYAASPAAAGYVTAGGYGYAVQQPITAAAPGTAAAA 212

C.elegans_SUP-12 -------------------------------RMGQVAG---------------------- 151

...

H.sapiens_RBM38 ------GTTFVQYQAPQLQPDRMQ 239

M.musculus_Rbm38 ------GTTFVQYQAPQLQPDRMQ 237

D.rerio_rbm38 -SAAAAAAAFGQYQPQQLQAERMQ 230

H.sapiens_RBM24 AAAAAAAAAFGQYQPQQLQTDRMQ 236

C.elegans_SUP-12 ------------------------
