# Supplementary material for: The RNA Binding Protein RBM38 (RNPC1) Regulates Splicing during Late Erythroid Differentiation
Source: PLoS One. 2013 Oct 18;8(10):e78031. doi: 10.1371/journal.pone.0078031 (PMC3820963; doi:10.1371/journal.pone.0078031)
Supplement: Table S3 — List of primer and oligonucleotide sequences used in this study. (DOCX) [file pone.0078031.s008.docx]

**Table S3**

**Primer name and sequence**

**RT-PCR**

KITLG F 5’-GCCCAGAACCCAGGCTCTTTACTC-3’

KITLG R 5’-AATGCCATGGCTGCCCAGTGT-3’

ISOC2 F 5’-TCGCCTACTTCCCACAGAT-3’

ISOC2 R 5’-GAGCAGGCGTCCACCACCACAT-3’

ERCC1 F 5’-CTGGGCGGTACCTGGAGACCTACA-3’

ERCC1 R 5’-ATCTTCTCTTGATGCGGCGATGAGC-3’

ZDHHC16 F 5’-CTTTTCCGGGAGGCTTATGCTGCC-3’

ZDHHC16 R 5’-TGCACAGGAACCAGAGGTAGA-3’

AP1G2 F 5’-TCGCGGGGCCAAGACTCAGG-3’

AP1G2 R 5’- CTTCTTGCGCACGTAGGGACTGG -3’

SIGMAR1 F 5’-GCCTTCTGCACGCCTCGCTGTCC-3’

SIGMAR1 R 5’-GCCCCGGCCGTACTCCACCATC-3’

GUSB F 5’-CGGCATTTTGTCGGCTGGGTGTG-3’

GUSB R 5’-TCCACGCTGGTGGTGACGGTGAT-3’

SFXN2 F 5’-CGATGCCGGCGGTGATCTTC-3’

SFXN2 R 5’-TCGCATCATGGGGATATTGACACA-3’

NRCAM F 5’-TTTCCAATGTCCTCCCAGAG-3’

NRCAM R 5’-CTTGCATTGCCTTCTGGAGT-3’

GOLGA2 F 5’- GGAATAGCCCTGGTGTTCCT-3’

GOLGA2 R 5’- CACGGTGTCATCAGATGGTT-3’

EPB41 F 5’-GCTGTCGATTCGGCAGACCGAAGTCCTCGGCCC-3’

EPB41 R 5’-TCCTGTGGGGATTTGCCCATTGATGTTAAG-3’

**Minigene**

4.1 Exon 13 F 5’-AGCCATTGCTCAGAGTCAGG-3’

4.1 Exon 17 R 5’-GCGAATTCCCGGATTCAGT-3’

PKC-F 5’-CCAGATGCCCGCAGCCCCACAA-3’

EGFP-R 5’-GGTCAGGGTGGTCACGAGGG-3’

Luc-R 5’-TATGTGCGTCGGTAAAGGCG-3’

**siRNA**

RBM38 #5 5’-AACCUGAAAGCAAGAAGUUAA-3’

**RT-PCR expression**

RBM24 F 5’-GGCAGAAAGGCCAACGTGAACTG-3’

RBM24 R 5’-CTGCGGTGATTGGCTGCTGGACT-3’

New actin F 5’-TGGGYGACGAGGCCCAGAGCA-3’

New actin R 5’-AGGTCCCGGCCAGCCAGGTCCAG-3’
